# Application of four pricing models for orphan medicines: a case study for lumasiran

**DOI:** 10.1186/s13023-024-03446-w

**Published:** 2024-12-23

**Authors:** Noa Rosenberg, Evert Manders, Sibren van den Berg, Lisa J. Deesker, Sander F. Garrelfs, Saco J. de Visser, Jaap W. Groothoff, Carla E. M. Hollak

**Affiliations:** 1 Medicine for Society, Platform at Amsterdam University Medical Center - University of Amsterdam, Amsterdam, The Netherlands; 2 Department of Endocrinology and Metabolism, Amsterdam UMC, University of Amsterdam, Amsterdam Gastroenterology Endocrinology Metabolism (AGEM) Research Institute, Meibergdreef 9, Amsterdam, The Netherlands; 3 Department of Pediatric Nephrology, Emma Children’s Hospital, Amsterdam University Medical Center - University of Amsterdam, Amsterdam, The Netherlands; 4 Centre for Future Affordable and Sustainable Therapy Development (FAST), The Hague, The Netherlands

**Keywords:** Pricing models, Lumasiran, Orphan medicinal products, Primary hyperoxaluria type 1

## Abstract

**Background:**

The combination of high prices and uncertain effectiveness is a growing challenge in the field of orphan medicines, hampering health technology assessments. Hence, new methods for establishing price benchmarks might be necessary to support reimbursement negotiations. In this study, we applied several pricing models containing cost-based elements to the case of lumasiran for treating primary hyperoxaluria type 1.

**Methods:**

Price ranges were calculated by estimating minimum and maximum scenarios for four pricing models: Novel Cancer Pricing Model (NCP-model), AIM Model for Innovative Medicines (AIM-model), Discounted Cash Flow model (DCF-model), and the Real-Option Rate Of Return model (ROROR-model). Data was gathered from disease registries, scientific literature, Security and Exchange Committee filings, and expert opinion. A sensitivity analysis was performed to assess the parameters with the largest influence.

**Results:**

Outcomes resulting from the NCP-model ranged between €87,000 and €224,000 per patient per year, between €33,000 and €340,000 for the AIM-model, between €182,000 and €748,000 for the DCF-model, and between €81,000 and €273,000 for the ROROR-model.

**Conclusion:**

Outcomes of the four pricing models show wide and heterogeneous price ranges. The DCF-model might be most compatible with the case of lumasiran, due to inclusion of parameters for prevalence, incidence, prescription restrictions and cost of capital. The minimum DCF price could serve as a starting point for pricing and reimbursement negotiations. Uncertainties can be solved by more transparency on input variables.

**Supplementary Information:**

The online version contains supplementary material available at 10.1186/s13023-024-03446-w.

## Introduction

An orphan medicinal product (OMP) is a medicine specifically developed for the treatment of a rare disease. In the European Union (EU), this pertains to diseases with a prevalence of less than 1 in 2000 [1]. Since 2000, incentives have been in place to stimulate the development of these products in the EU, including discounts on scientific advice and marketing authorization applications, as well as a 10-year market exclusivity (monopoly position) [1].

While the authorization of OMPs proceeds via the centralized procedure in the EU, reimbursement has to be arranged on the level of individual EU Member States. Thus, many Member States have established so-called health technology assessment (HTA) bodies, which are responsible for providing advice and/or decisions regarding reimbursement. Cost-effectiveness analyses are often conducted by these HTA-bodies to support informed decision-making [2, 3]. These analyses evaluate both costs and health effects, assessing the costs of a gained health unit (i.e. year of life). [2, 3]. Following these procedures, negotiations frequently take place between the marketing authorization holder and payers or governments in an undisclosed fashion [4].

There are limitations to this strategy, particularly in the field of rare diseases. First, the outcomes of cost-effectiveness analyses based on value-based prices often exceed national reimbursement thresholds. Additionally, these analyses may have to rely on numerous assumptions based on limited effectiveness data at the time of market entry [5]. This paucity of effectiveness data is frequently a consequence of inherent rarity of the disease, leading to an increasing number of conditional approvals and approvals under exceptional circumstances [6]. Consequently, post-approval studies are often mandatory [6]. However, at the same time, national HTA-bodies, payers, policymakers, society, and politicians experience increased resistance to paying prices above national reimbursement thresholds for medicines with uncertain effectiveness in the real-world setting. This can complexify the negotiations and impede fast and managed access.

Notably, OMPs are generally higher priced than non-OMPs [7]. This may be explained by several aspects, including costs that have to be recouped over small patient populations and the value-based pricing strategy. This strategy involves determining a price that reflects health benefits of the treatment compared to the standard care [8, 9]. Introducing innovative access pathways requires tools, such as novel pricing models that could provide new pricing benchmarks when value-based pricing is unsuitable. This could be particularly relevant for payers when a cost-effectiveness analysis is deemed unfeasible because of high uncertainties on clinical effectiveness.

Recently, a number of pricing models that contain cost-based elements have been developed [10–16]. These models could promote prices based on the recoupment of costs, rather than relying on uncertain value-based pricing. The feasibility of calculating cost-based prices relies mainly on the availability of prevalence data and on the transparency of costs by the manufacturer. Several of these models have already been applied and documented, featuring innovative gene therapy products such as nusinersen and onasemnogene, as well as biological products like pertuzumab and the repurposing of an old medicine, mexiletine [11, 12, 16, 17].

The goal of this paper is to apply a variety of pricing models with cost-based elements to a case study of an innovative product in the EU. We purposively selected the RNAi-based lumasiran for the treatment of primary hyperoxaluria type 1 (Box 1) due its innovative nature and our hospitals involvement in establishing the managed entry agreements. Accordingly, we calculated price ranges based on minimum and maximum scenarios. Finally, we discuss potential applications of pricing models containing cost-based elements as tools to calculate benchmark prices for innovative OMPs.

**Box 1:** Lumasiran for the treatment of primary hyperoxaluria type 1

Lumasiran (trade name OXLUMO™, Alnylam® Pharmaceuticals) is a novel RNAi therapy developed for the treatment of primary hyperoxaluria type 1 (OMIM #259900, PH1) in all age groups [18, 19]. PH1 is a rare disease that affects approximately 1.6–2.9 per 1 million people [20, 21]. It is caused by a mutation in the *AGXT* gene that results in a deficit of the hepatic enzyme alanine-glyoxylate aminotransferase (AGT) [22–24]. The reduced enzyme activity leads to increased levels of oxalate, resulting in calcium oxalate deposits [25]. PH1 is characterized by heterogenous clinical phenotypes, ranging from occasional kidney stones to end-stage renal disease (ESRD) and concurrent life-threatening systemic disease within the first years of life. Despite this heterogeneity, more than 70% of patients will eventually develop ESRD at some point in their life [23, 26]. A minority of patients responds well to pyridoxine (vitamin B6) treatment, while the majority is dependent on burdensome care including dialysis and liver and/or kidney transplantation [26]. Lumasiran has shown to be highly effective in reducing endogenous oxalate production in PH1 patients.	

## Methods

We selected four pricing models containing cost-based elements with value-driven bonuses based on a systematic review of scientific and grey literature [69] and applied them to the case of lumasiran. Input parameters of each model are summarized in Table 1. An extensive explanation of each model can be found in Additional files 1–4. The models adopt different perspectives:

1. Novel cancer price model (NCP-model): This model combines both cost-based as well as value-based elements. It focuses on transparency by requiring precise data on various costs elements. It anticipates on clinical value, by including a profit margin that is linked to the anticipated level of clinical benefit [15].
2. AIM model for “fair and transparent prices for accessible pharmaceutical innovations” (AIM-model): the AIM-model has a similar approach to the NCP-model, but introduces assumptions to estimate the costs. This provides flexibility and can accommodate pricing calculation with a restricted level of cost data. The model provides various lump sums for R&D costs based on literature, some of which include the Cost of Capital (CoC). CoC represents the minimum return before a project generates any value, accounting for the costs of debt and equity. The variable inclusion of CoC likely leads to a wide range of lump sums, starting at €250 million. Higher costs are possible by demonstrating actual R&D costs but are capped at €2.5 billion, whether or not CoC are included. Furthermore, it includes factors, such as a market share, to estimate the number of patients actually receiving treatment. An innovation bonus encourages innovation, but also introduces uncertainty as it is linked to potential clinical benefits [14].
3. Discounted cash flow model (DCF-model): The DCF-model utilizes a different approach from the AIM- and NCP-model as it is grounded in financial theory, focusing on achieving a return on investment from the investor’s perspective. The DCF-model calculates a price at which the manufacturer reaches the break-even point at the time of the patent expiry [13]. By introducing the cost of capital as a discount factor, it ensures that the future cash flows are sufficient to cover the investments and provide the expected return. In contrast to the NCP- and AIM-model, the DCF-model uses lump sums which do not promote cost transparency in more detail.

In a more recent paper, Nuijten & Capri proposed adding an innovation premium as a surplus to the break-even price, consisting of the monetary value for gained quality-adjusted life years (QALYs) and cost savings [11]. However, for the lumasiran case, we consider these elements too uncertain and chose to include a fixed percentage of 5 to 15 percent as an innovation premium for the company [13].

4. Real-option rate of return model (ROROR-model): The ROROR-model incorporates elements of real options theory, which considers uncertain investment decisions. It aims to establish transparent medicine prices to both healthcare systems and patients, while maintaining sufficient incentives for innovation within the industry. This entails a more thorough assessment of costs associated with R&D than the NCP-, AIM- and DCF-model, factoring in opportunity, out-of-pocket, failure, and CoC and considering resource allocation and time separately [27]. Furthermore, to address global economic disparities, the model adopts a worldwide differential pricing strategy, adjusting prices based on the gross domestic product (GDP) in different countries or regions. The model then computes the price per patient per year (ppppy).

**Table 1:** Overview of input variables in various pricing models

Input variable	Novel cancer pricing model	AIM model	Discounted cash flow model	Real-option-rate-of return model
R&D costs*	x	x	x	x
Out of pocket costs				x
Failure costs*	Included in R&D costs	Included in R&D costs	Included in R&D cost	x
Cost of capital*			x	x
GDP deflator				x
Drug cost			x	
Manufacturing costs*	x	x		
Marketing and sales costs*	x	x		
Patent years*	x	x	x	x
Average time until approval				x
Number of patients	x	x		x
Prevalent patients*			x	
Incident patients*			x	
Population growth			x	
Treatment rate*	x	x		
Treatment response rate			x	
Treatment failure rate			x	
Adverse event rate			x	
Contraindication rate			x	
Prescription restrictions*			x	
Market share*		x	x	
Profit margin	x	x		x
Innovation bonus		x	x +	

The models include the Novel cancer pricing model, the AIM model, Discounted cash flow model, and the Real-option-rate-of return model

R&D, Research and development; GDP, Gross domestic product

*Particularly relevant for the case of lumasiran for the treatment of primary hyperoxaluria type 1

+ In our application we choose to use a fixed percentage as an innovation premium instead of QALYs and cost savings because this was deemed too uncertain

When comparing the abovementioned pricing models, it is important to highlight both their similarities and distinctions. Notably, the ROROR-model, alongside the NCP- and AIM-model, incorporate relatively similar cost-based elements. However, the AIM-model introduces treatment rates and market shares as additional components, potentially providing a more precise estimate of the actual number of patients receiving treatment. Another difference is that the NCP-model demands more actual cost data compared to the AIM-model, the latter relying more on assumptions regarding incurred costs. Moreover, variations exist in predetermined assumptions. For instance, the AIM-model combines an 8% profit margin with an innovation bonus ranging from 0 to 40%, contrasting with the NCP-model’s profit margin range of 10–30%. A thorough exploration of these distinctions is available in Additional files 1 and 2.

The DCF- and ROROR-model significantly diverge in approach from the NCP- and AIM-model, as these models take a broader perspective on cost-based elements. They not only consider R&D or production costs but also explicitly account for the required CoC. This inclusion considers the investor’s perspective, aiming to achieve a return on investments. The DCF-model, in particular, employs more specific components for calculating the number of patients per year and applies a discount rate to each year’s cash flows. Comprehensive details on the DCF- and ROROR-model can be found in Additional files 3 and 4.

Some models use foreign currencies or global costs. To reflect a European context, U.S. dollars were converted to Euros using the the 2021 exchange rate average [28]. Furthermore, we calculated the R&D costs attributable to the EU by utilizing the proportion of the EU population over the total population in developed countries (35.85%) for all models [14, 29].

### Pricing scenarios

To cover uncertainties, we established minimum and maximum scenarios by estimating the minimum and maximum input values for the EU market, as outlined in Tables 2, 3, 4 and 5. The AIM- and DCF-model offer standardized assumptions for small molecules and biologicals/biotech [13, 14]. Since RNAi therapy does not fit either category, we opted for the small molecule and biological assumptions for the minimum and maximum scenarios, respectively.

**Table 2:** Minimum, maximum and average scenarios for the novel cancer pricing model

Input variable	Minimum scenario	Maximum scenario	Average scenario
**Eligible patients**	**907**	**359**	**604**
Prevalence	2.9:1 million [20]	1.6:1 million [21]	2.25:1 million
Treatment rate	70% [30]	50% [24, 31]	60%
EU population	447 million [32]	447 million [32]	447 million
**Patent years left**	**14** [33]	**10** [34]	**12**
**R&D costs PPPY**	** ~ €23 k**	**~ €111 k**	**~ €48 k**
Average R&D costs per approved Alnylam medicine	~ €830 million [33, 35–52]	~ €1.1 billion [33, 35–52]	~ €970 million
Percentage attributable to EU	35.85% [14]	35.85% [14]	35.85%
**Drug costs PPPY**	**~ €49 k**	**~ €49 k**	~ €49 k
Cost of sold goods PPPY (i.e. the costs of producing the goods that are sold by the company)	~ €38 k [33, 43, 44]	~ €38 k [33, 43, 44]	~ €38 k
Marketing and Sales PPPY	30% of manufacturing [15]	30% of manufacturing [15]	30% of manufacturing
**Profit margin**	**20%** [15]	**40%** [15]	**30%**
**Average price per patient per year**	**~ €87 k**	**~ €224 k**	** ~ €126 k**

The model was applied to the case of lumasiran for the treatment of primary hyperoxaluria type 1. The calculations based on the input variables lead to an average price per patient per year. If a specific factor is inversely proportional to the outcome, the highest number represents the minimum scenario, and vice versa

PPPY, Per patient per year

**Table 3:** Minimum, maximum and averages scenarios for the AIM model

Input variable	Minimum scenario	Maximum scenario	Average scenario
**Eligible patients**	**907**	**179**	**452**
Prevalence	2.9:1 million [20]	1.6:1 million [21]	2.25:1 million
Treatment rate	70% [30]	50% [30, 31]	60%
Market share	100%	50%	75%
EU population	447 million [32]	447 million [32]	447 million
**Patent years left**	**14** [33]	**10 **[34]	**12**
**R&D costs PPPY**	**~ €22 k**	**~ €222 k**	** ~ € 63 k**
Average R&D costs per approved Alnylam medicine	~ €800 million [14]	~ €1.1 billion [33, 35–52]	~ €954 million
Percentage attributable to EU	35.85% [14]	35.85% [14]	35.85%
**Production and overhead PPPY**	** ~ €3 k** [14]	** ~ €9 k** [14]	** ~ €6 k**
**Sales and medical information PPPY**	**15% of R&D** [14]	**20% of R&D costs** [14]	**18% of R&D costs**
**Basic profit**	**8%** [14]	**8%** [14]	**8%**
**Innovation bonus**	**5%** [14]	**40%** [14]	**23%**
**Average price per patient per year**	**~ €33 k**	**~ €400 k**	**~ €104 k**

The model was applied to the case of lumasiran for the treatment of primary hyperoxaluria type 1. The calculations based on the input variables lead to an average price per patient per year. If a specific factor is inversely proportional to the outcome, the highest number represents the minimum scenario, and vice versa

R&D, research and development; PPPY, per patient per year

**Table 4:** Minimum, maximum and average scenarios for the discounted cash flow model

Input variable	Minimum scenario	Maximum scenario	Average scenario
**Average number of eligible patients**	**387**	**186**	**253**
Prevalence	2.9:1 million [20]	1.6:1 million [21]	2.25:1 million
Incidence	0.15:1 million [20]	0.18:1 million [21]	0.17:1 million
Response rate	100% [19, 53–56]	95% [19, 53–56]	97.5%
Failure rate	0% [19, 53–56]	5% [19, 53–56]	2.5%
Adverse events	0% [19, 53–56]	5% [19, 53–56]	2.5%
Contraindications	0% [19, 53–56]	5% [19, 53–56]	2.5%
Prescription restrictions prevalent population	LTx 30% [30] Fully pyridoxine responsive 15% [31]	LTx 30% [30] Fully pyridoxine responsive 15% [31] Additional 5% because some partially pyridoxine responsive might not receive treatment	47.5%
Prescription restrictions incident population	Fully pyridoxine responsive 15% [31]	Fully pyridoxine responsive 15% Additional 5% because some partially pyridoxine responsive might not receive treatment	17.5%
EU population	447 million [32]	447 million [32]	447 million
EU population growth	0%	1% [57]	0.5%
Uptake	Max 50%	Max 50%	Max 50%
**Patent years left**	**14** [33]	**10** [34]	**12**
**R&D costs**	**~ €228 million**	**~ €228 million**	** ~ €228 million**
Lumpsum R&D costs	~ €637 million [11]	~ €637 million [11]	~ €637 million
Percentage attributable to EU	35.85%	35.85%	35.85%
**Drug costs**	**40% of revenues** [11]	**40% of revenue** [11]	**40% of revenue**
**Cost of capital**	**9%** [11]	**12%** [11]	**10.5%**
**Break even price**	** ~ €173 k**	**~ €650 k**	**~ €342 k**
**Innovation premium**	**5%**	**15%**	**10%**
**Average price per patient per year**	** ~ €182 k**	**~ €748 k**	**~ €377 k**

The model was applied to the case of lumarisan for the treatment of primary hyperoxaluria type 1. The calculations based on the input variables lead to an average price per patient per year. If a specific factor is inversely proportional to the outcome, the highest number represents the minimum scenario, and vice versa

LTx, Has received liver transparantation; R&D, Research and development

**Table 5:** Minimum, maximum and average scenarios for the real-option rate of return model

Input variable	Minimum scenario	Maximum scenario	Average scenario
**Eligible patients**	**907**	**359**	**604**
Prevalence	2.9:1 million [20]	1.6:1 million [21]	2.25:1 million
EU population	447 million [32]	447 million [32]	447 million
**Patent years left**	**14** [33]	**10** [34]	**12**
**Production costs**	**~ €38 k** [33, 43, 44]	** ~ €38 k** [33, 43, 44]	** ~ €38 k**
**R&D costs PPPY**	**~ €23 k**	**~ €111 k**	**~ €48 k**
Average R&D costs per approved Alnylam medicine	~ €830 million [33, 35–52]	~ €1.1 billion [33, 35–52]	~ €970 million
Percentage attributable to EU	35.85% [14]	35.85% [14]	35.85%
**WACC%**	**8%**	**14%**	**11%**
**GDP deflator**	**1.9%**	**6.5%**	**4.2%**
**Time until approval**	**6** [58]	**15** [59]	**9.5**
**Profit margin**	**5%** [15]	**15% **[15]	**30%**
**Average price per patient per year**	** ~ €81 k**	**~ €273 k**	** ~ €134 k**

The model was applied to the case of lumarisan for the treatment of primary hyperoxaluria type 1. The calculations based on the input variables lead to an average price per patient per year. If a specific factor is inversely proportional to the outcome, the highest number represents the minimum scenario, and vice versa

R&D, Research and development; PPPY, Per patient per year; WACC, Weighted average cost of capital; GDP, Gross domestic product

Data concerning the number of pediatric and adult patients were gathered from scientific literature, Eurostat, the OxalEurope Registry, and the European Public Assessment Report (EPAR). Following the suggestion of Prasad & Mailankody, United States (US) Security and Exchange Committee (SEC) filings were used for actual costs data [60]. These files were accessed through the public EDGAR search database [61]. Aggregated costs from the SEC filings were divided by the total number of approved products, thereby incorporating the cost of failed projects (See Additional file 5 for detailed data and calculations).

Next, estimates for minimum and maximum scenarios were made based on the collected data for each input variable and interpreted with the help of expert opinion (Tables 2, 3, 4 and 5; Additional files 6, 7). Average scenarios were formulated by averaging the minimum and maximum values of each input parameter. For both the minimum and maximum scenario, an analysis was performed to determine the contribution of individual input parameters to the total price per patient per year (ppppy). The final calculated ppppys were rounded to the nearest thousand.

### Sensitivity analysis

To examine the impact of input parameters on the calculated ppppy, a sensitivity analysis was conducted to all models. This involved exploiting the average scenario and substituting each individual input parameter with both the respective minimum and maximum estimates.

To assess the impact of each model’s strategy on estimating the actual number of patients receiving treatment, a supplementary analysis was conducted by inserting 1000 patients for both the minimum and maximum scenario. During this analysis, input factors such as treatment rate, market shares and uptake curves were disregarded.

## Results

Price ranges for the case of lumasiran were derived from minimum and maximum scenarios containing estimates for each input variable. Average scenarios were created by calculating the mean of the minimum and maximum input values. Table 1 offers an outline of input variables per pricing model, while Tables 2, 3, 4 and 5 provide an overview of scenarios. Detailed descriptions are available in the Additional files.

**Fig. 1:**
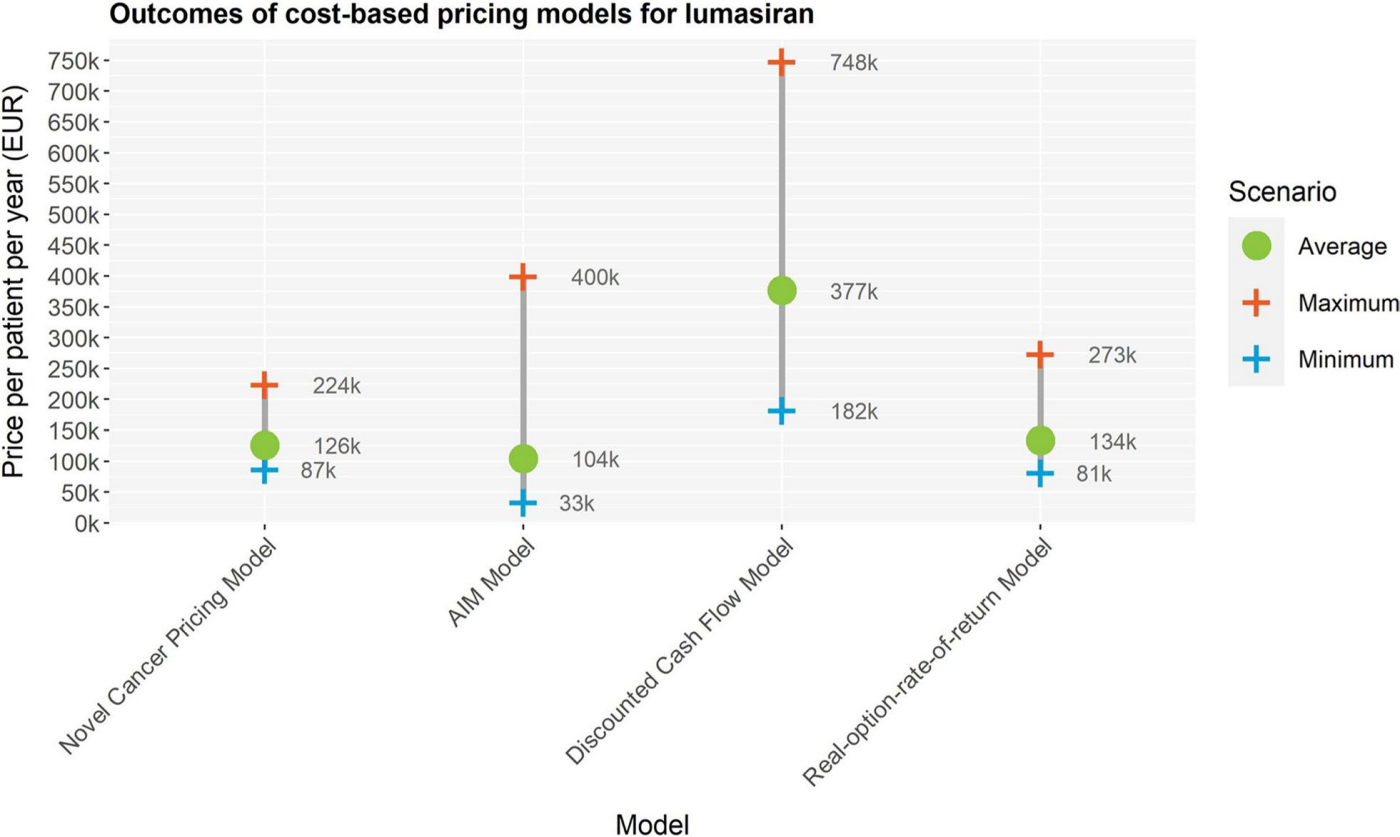
Overview of pricing outcome ranges. Price ranges are displayed for the Novel cancer pricing model, AIM model and Discounted cash flow model and real-option rate of return model based on minimum, maximum and average scenarios for the case of lumarisan for the treatment of primary hyperoxaluria type 1

Price ranges differ across pricing models (Fig. 1). The scenarios revealed the following ppppy, for a patient with an average weight:

- NCP-model: €87,000–€224,000 (average scenario €126,000)
- AIM-model: €33,000–€400,000 (average scenario €104,000)
- DCF-model: €182,000–€748,000 (average scenario €377,000)
- ROROR-model: €81,000–€ 273,000 (average scenario €134,000)

The models incorporating more input parameters exhibit wider price ranges. A price range of €182 000 to €224 000 spans across all outcomes.

**Fig. 2:**
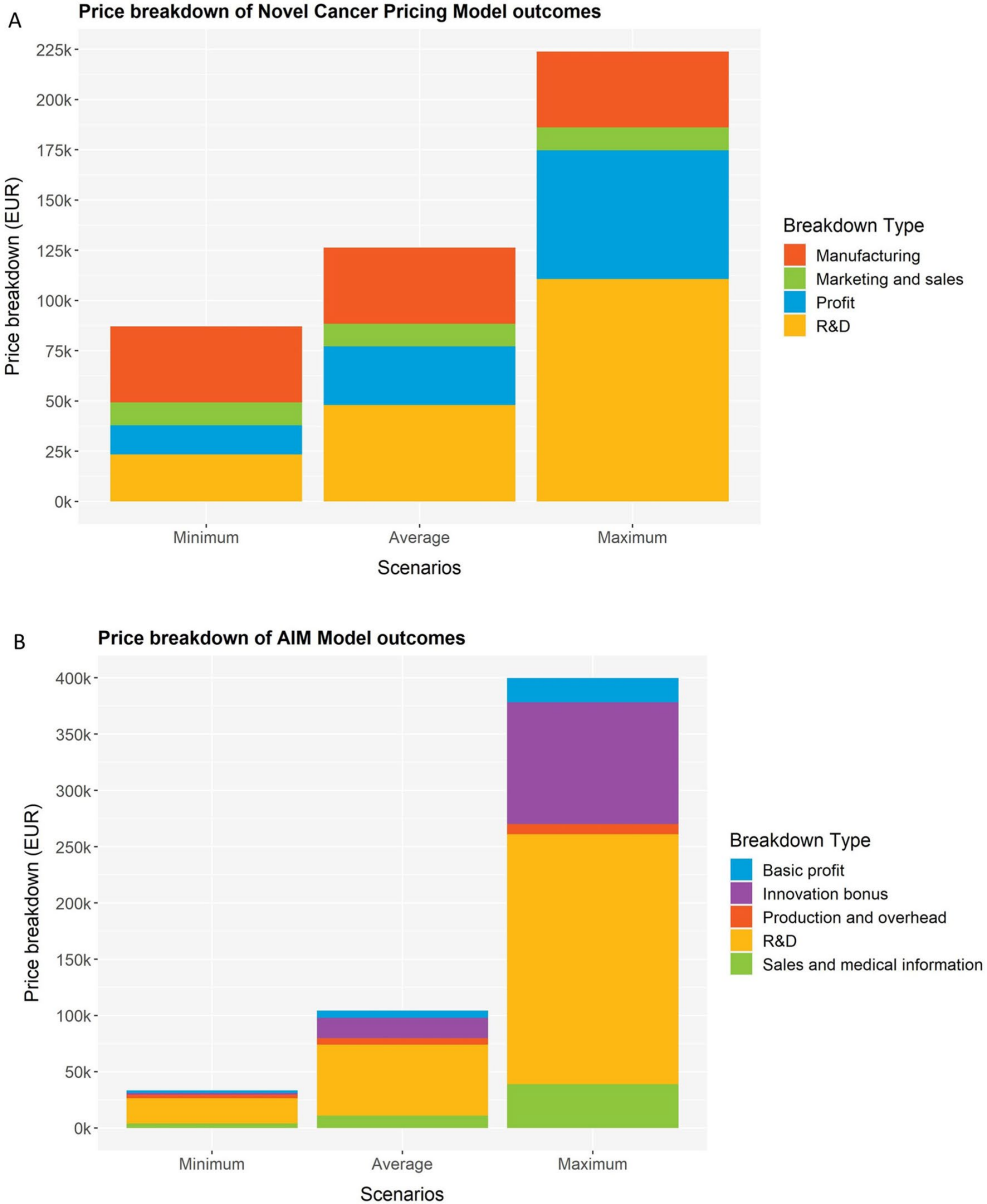

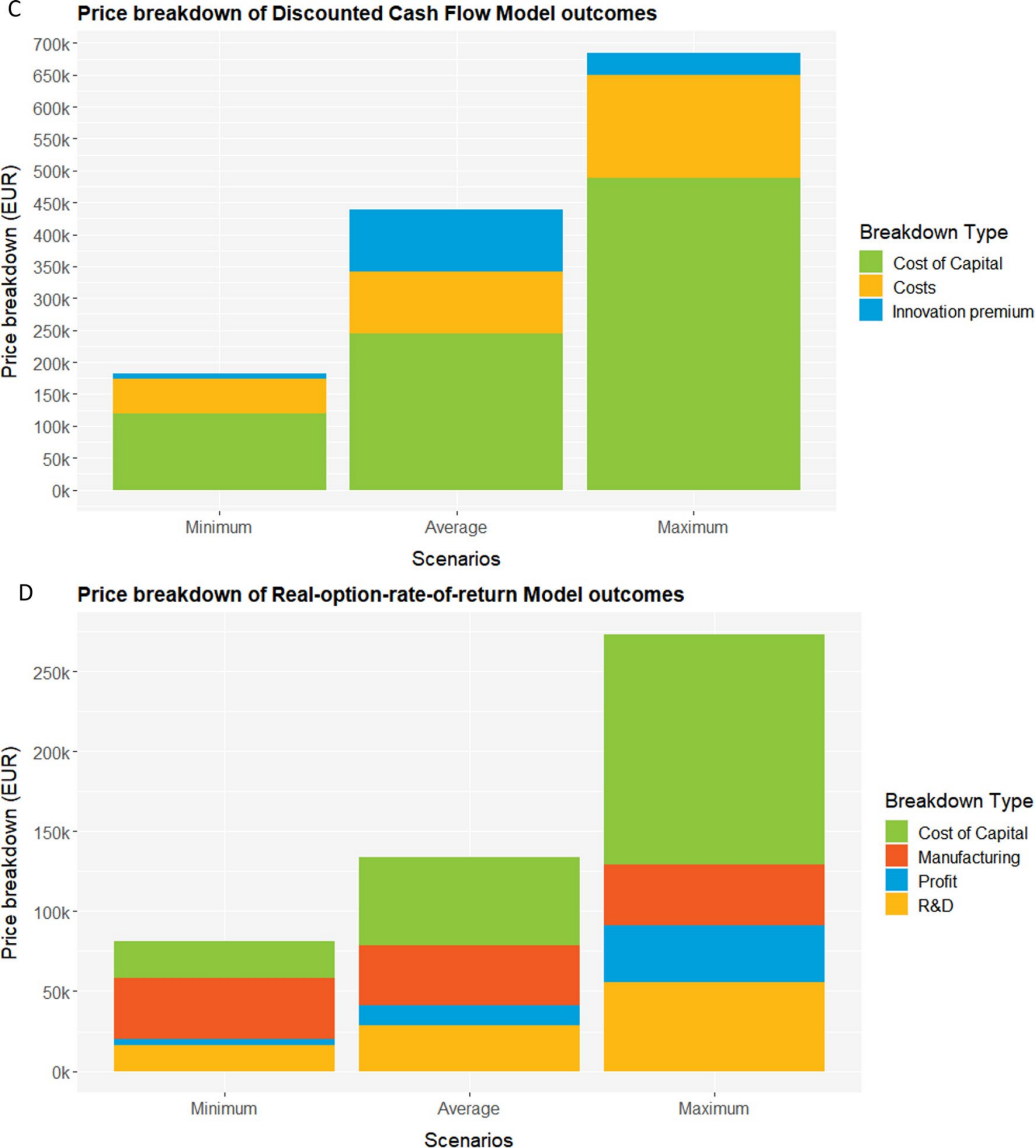
Overview of price breakdown for minimum, maximum and average scenarios for the four pricing models. The pricings models were applied to the case of lumasiran for the treatment of primary hyperoxaluria type 1. **A** Novel cancer pricing model, **B** AIM model, **C** Discounted cashflow model. **D** Real-option rate of return model

Examining the price breakdown (Fig. 2), R&D costs are the largest proportion in the NCP- and AIM-model outcomes. For the DCF- and ROROR-models, the largest proportion represents the CoC.

### Sensitivity analysis

**Fig. 3:**
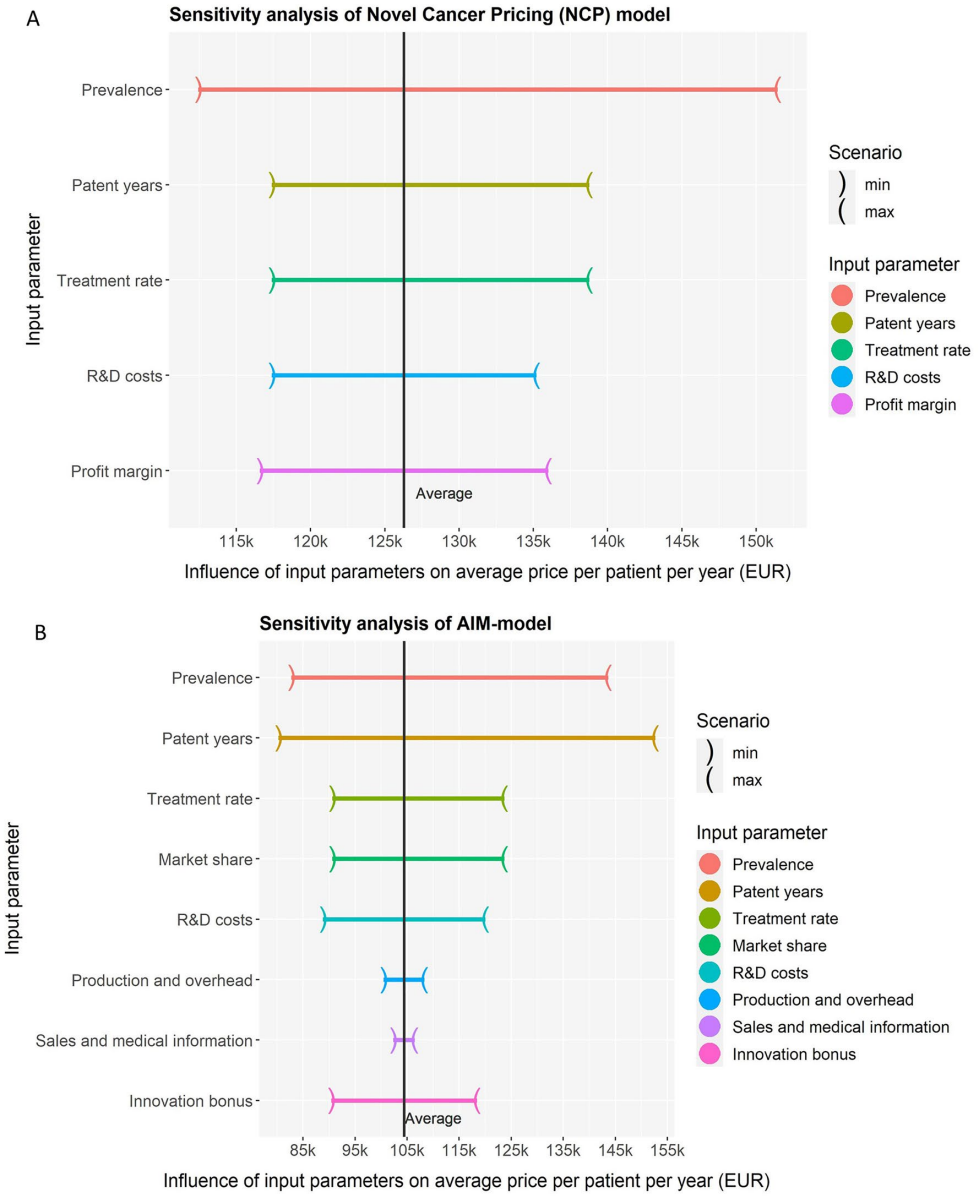

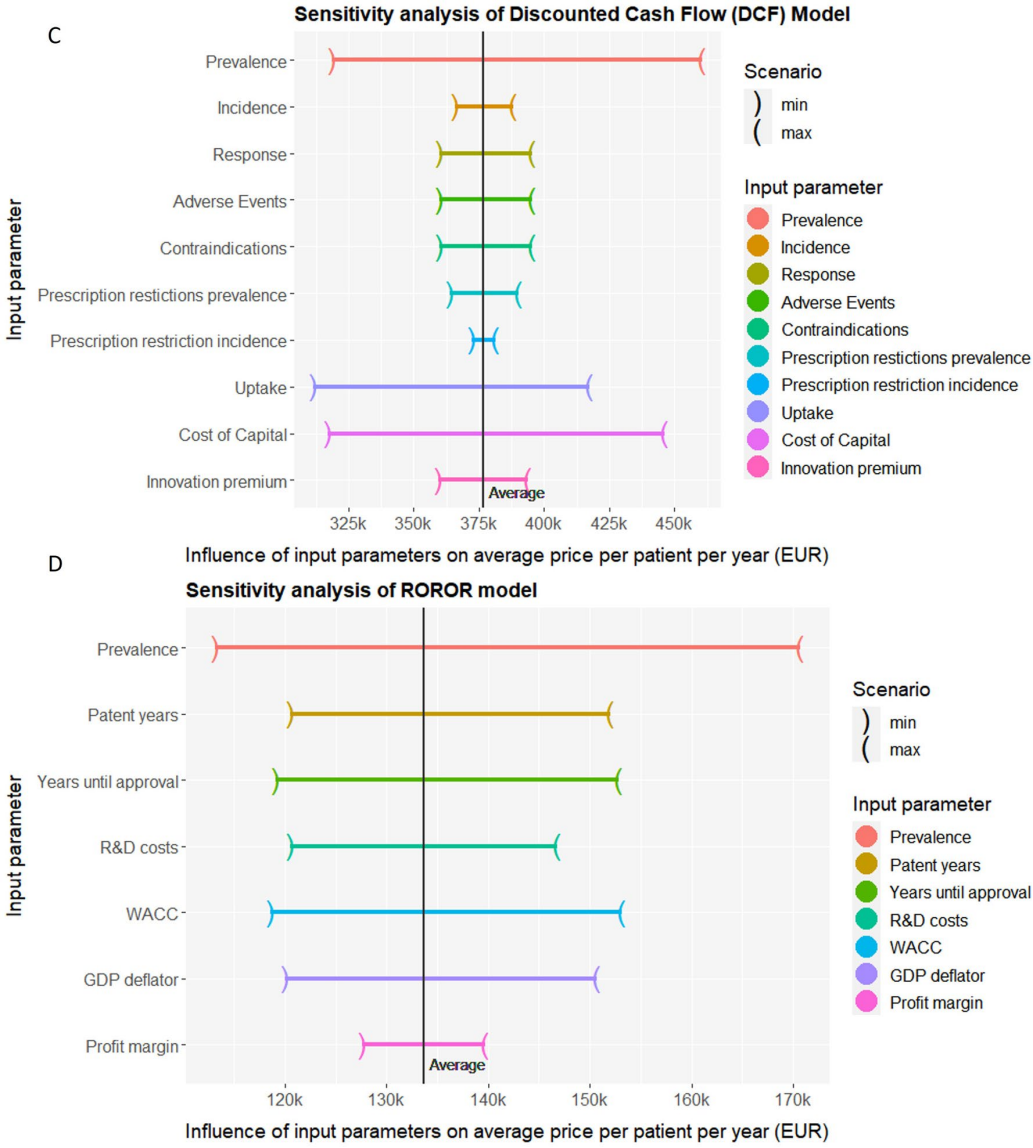
Sensitivity analysis of the for pricing models applied to the case of lumasiran. The plots illustrate the variation in outcomes when the input values of the average scenario were substituted with the minimum and maximum values of each corresponding individual input parameter. The vertical line represents the average outcome of each pricing model. **A** Novel Cancer Price Model, **B** AIM model, **C** Discounted Cash Flow model, and **D** Real-option-rate-of-return model

To better understand the impact of different variables in the pricing models, we conducted a sensitivity analysis. This analysis assesses how variations in the input parameters of the average scenario influence the pricing outcomes. This analysis reveals that the prevalence parameter is very sensitive across all models (Fig. 3), potentially due to uncertainty on the estimated number of patients emphasizing the importance of accurate prevalence estimates. Besides, the AIM-model is predominantly influenced by the remaining patent years. Length of time remaining on the patent affect the overall pricing outcomes, but can be accurately determined. Moreover, the CoC has a large influence on the DCF-pricing outcomes. Small variation in the CoC lead to substantial changes in the pricing outcomes underscoring the DCF-model’s reliance on accurate CoC estimates. These findings emphasizes the substantial impact changes and uncertainty on input variables have on pricing outcomes.

A supplementary analysis revealed that the strategy for determining the number of patients actually receiving patients impacts the outcomes (Additional file 8). When the number of patients is kept constant for each model, and factors such as treatment rates and market shares are disregarded, models incorporating many for these factors, such as the DCF-model, yield lower prices.

## Discussion

In this study, we calculated price ranges of lumasiran using four pricing models containing cost-based elements [11, 12, 16, 17, 62]. The results demonstrate substantial deviations in price ranges among the models, primarily due to inherent differences in underlying assumptions, inclusion of extra input variables, or requirement of actual costs instead of lump sum values. If we compare the outcomes to the weight-dependent German list price of lumasiran of €313,940 to €941,822 per patient per year, we observe that the lower end falls within the AIM and DCF price range, but the upper end exceeds it [19, 63].

Lumasiran for the treatment of PH1 is Alnylam Pharmaceuticals’s third authorized RNAi therapy [33], marking its development as both innovative and risky. Risky projects often involve higher CoC, making it an important factor to consider for this case. Therefore, some models may be less fitting. For instance, the NCP-model does not directly account for CoC, making it less compatible. Similarly, the AIM-model’s application of CoC is uncertain, as it is ambiguous on whether CoC are included in its R&D lump sums, potentially explaining its wide range [64]. Although the AIM-model encourages transparency by allowing for additional R&D expenditures based on actual costs, it does not directly incorporate CoC.

In contrast to the NCP- and AIM-model, both the DCF- and ROROR-model clearly incorporate CoC. However, the ROROR-model lacks certain elements, such as sales costs, market shares, and innovation bonuses, which could result in underestimations. Besides, the application of the ROROR-model proved questionable due to the limited guidance on determination of costs and patient numbers.

Given that the NCP-, AIM-, and ROROR-model appear to be less appropriate for lumasiran and offer less guidance, the DCF-model emerges as the most suitable pricing model for this case. This model is advantageous for several reasons. First, the DCF-model explicitly account for CoC, which is crucial for reflecting the expectations of investors. Second, the DCF-model utilizes multiple parameters to estimate the actual number of patients receiving treatment. These factors have a large influence on cost-based pricing outcomes. For lumasiran specifically, incorporating prevalence, incidence and prescription restrictions are appropriate, considering factors such as liver transplantations and full or partial pyridoxine responsiveness. Last, the uptake curve might be legitimate to cover for the gradual market entry of lumasiran and potential competing treatments, such as nedosiran, another RNAi treatment that has shown similar efficacy in a randomized clinical trial [65, 66]. These factors makes the DCF-model more precise in estimating potential revenues.

A downside of the DCF-model is parameter uncertainty. This model contains many parameters and uncertainty regarding the estimated input variables for each of these parameters results in a wide price range. To overcome this, the minimum DCF-scenario could be used as a start to establish a benchmark price for initiating price negotiations. By providing transparency on the actual price buildup and incurred costs, the company could justify a higher price. This may be particularly important since CoC, a major driver of the price in this model, may consist of several undisclosed elements including high return on investments for shareholders. Besides, to what extent health payers should accommodate investors’ return requirements remains a political question.

As the inadequacy of cost-effectiveness analyses for OMPs becomes apparent due to lack of insights on clinical value, exploring what pricing model containing cost-based elements could be most suitable to what case becomes imperative. Adapting pricing strategies to accommodate new circumstances over time may become necessary. Upon establishing clinical effectiveness, incorporating value-based components could be considered. Finally, when sufficient return on investments have been achieved, the suitability of models integrating CoC should be reexamined, as production plus profit pricing models might become more applicable [67]. However, the implementation of these changes requires coorperation from industry and a more dynamic reimbursement system.

Several limitations must be acknowledged regarding the application of the pricing models to the case of lumasiran. Whilst we created minimum and maximum scenarios per pricing model to address uncertainties of our input values, outcomes must be interpreted cautiously as the implementation of scenarios potentially led to under- or overvaluation. Regarding cost aspects, actual cost data were gathered from Alnylam Pharmaceuticals’ US SEC filings, offering aggregated costs with a relatively limited level of detail potentially leading to overestimations (Table 2).

Considering the number of patients, the prevalence and incidence might have been underestimated due to inadequate diagnosis. The availability of a treatment could lead to more frequent diagnostic testing, which may increase the observed prevalence of the disease [23, 24, 26]. Moreover, start-stop criteria may vary across EU Member States, impacting the prescription of lumasiran for subgroups (pyridoxine responsiveness and/or liver transplantation). Last, the application of the pricing models resulted in a price based on the number of patients in the EU, without accounting for differentiation per member state, number of vials, body weight, spillage or other matters. Some of these uncertainties could be mitigated by managed entry agreements that include phased implementation and enhanced data collection through registers.

New iterations of pricing models may include additional cost elements, such as risk discounts or premiums [13]. To avoid wide price ranges in future applications of cost-based pricing models, refinement of estimation methodologies for input variables is key. Standardized methods for calculating and communicating actual R&D costs, costs of failed projects, and CoC should be implemented by industry. A first attempt has been drafted by Médicins Sans Frontières regarding clinical trial costs transparency [68]. Furthermore, better estimations of the number of patients that will actually receive treatment are essential to more defined price ranges.

## Conclusion

New methods for establishing price benchmarks might be necessary to support reimbursement negotiations, as health technology assassments for OMPs provide insufficient insights due to paucity of effectiveness data. The results of the four pricing models containing cost-based elements exhibit heterogeneous price ranges. The DCF-model appears to be the most suitable for lumasiran for the treatment of PH1, as it incorporates parameters such as prevalence, incidence, prescription restrictions, and cost of capital. The minimum DCF price could provide a foundation to support pricing and reimbursement discussions. To prevent wide price variations in future applications of cost-based pricing models, it is essential to refine the estimation of input variables.

## Supplementary Information

Additional file1 (DOCX 90 KB)

## Data Availability

The dataset(s) supporting the conclusions of this article is(are) included within the article (and its additional file(s)).

## Abbreviations

AGT: Enzyme alanine-glyoxylate aminotransferase
AIM-model: AIM model for “fair and transparent prices for accessible pharmaceutical innovations”
CoC: Cost of Capital
DCF-model: Discounted cash flow model
EPAR: European Public Assessment Report
ESRD: End-stage renal disease
EU: European Union
GDP: Gross domestic product
HTA: Health technology assessment
NCP-model: Novel cancer price model
OMP: Orphan medicinal product
PH1: Primary hyperoxaluria type 1
ppppy: Price per patient per year
QALYs: Quality-adjusted life years
R&D: Research and development
RoR: Rate of return
ROROR-model: Real-option rate of return model
SEC: Security and Exchange Committee
US: United States

## References

[1] European Medicines Agency. Orphan incentives. n.d. Available from: https://www.ema.europa.eu/en/human-regulatory/research-development/orphan-designation/orphan-incentives. Accessed 3 Feb 2023.

[2] Centers for Disease Control and Prevention. Cost-effectiveness analysis. 2021. Available from: https://www.cdc.gov/policy/polaris/economics/cost-effectiveness/index.html. Accessed 3 Feb 2023.

[3] Office for Health Improvement and Disparities. Cost effectiveness analysis: health economic studies. 2020. Available from: https://www.gov.uk/guidance/cost-effectiveness-analysis-health-economic-studies. Accessed 3 Feb 2023.

[4] Simoens S. Pricing and reimbursement of orphan drugs: the need for more transparency. Orphanet J Rare Dis. 2011;6:42. 21682893 10.1186/1750-1172-6-42 PMC3132155

[5] Deticek A, Locatelli I, Kos M. Patient access to medicines for rare diseases in European countries. Value Health. 2018;21(5):553–60. 29753352 10.1016/j.jval.2018.01.007

[6] Jonker CJ, et al. Registries supporting new drug applications. Pharmacoepidemiol Drug Saf. 2017;26(12):1451–7. 28983992 10.1002/pds.4332 PMC5725674

[7] Picavet E, et al. Shining a light in the black box of orphan drug pricing. Orphanet J Rare Dis. 2014;9:62. 24767472 10.1186/1750-1172-9-62 PMC4018963

[8] Raftery J. Value based pricing: Can it work? BMJ. 2013;347: f5941. 24124158 10.1136/bmj.f5941

[9] Hughes DA. Value-based pricing: Incentive for innovation or zero net benefit? Pharmacoeconomics. 2011;29(9):731–5. 21736392 10.2165/11592570-000000000-00000

[10] Berdud M, Drummond M, Towse A. Establishing a reasonable price for an orphan drug. Cost Eff Resour Alloc. 2020;18:31. 32908456 10.1186/s12962-020-00223-x PMC7472708

[11] Nuijten M, Capri S. Pricing of orphan drugs in oncology and rare diseases. J Mark Access Health Policy. 2020;8(1):1838191. 33312455 10.1080/20016689.2020.1838191 PMC7717868

[12] van den Berg S, et al. Cost-based price calculation of mexiletine for nondystrophic myotonia. Value Health. 2021;24(7):925–9. 34243835 10.1016/j.jval.2021.02.004

[13] Nuijten M, Vis J. Evaluation and valuation of innovative medicinal products. J Rare Dis Res Treat. 2016;2(1):1–11.

[14] The International Association of Mutual Benefit Societies (AIM), AIM proposes to establish a European drug pricing model for fair and transparant prices for accessible pharmaceutical innovations. 2019, The International Association of Mutual Benefit Societies. Available from: https://www.aim-mutual.org/wp-content/uploads/2019/12/AIMs-proposal-for-fair-and-transparent-prices-for-pharmaceuticals.pdf. Accessed 21 July 2022.

[15] Uyl-de Groot CA, Löwenberg B. Sustainability and affordability of cancer drugs: a novel pricing model. Clin Oncol. 2018;15:405–6. 10.1038/s41571-018-0027-x 29735987

[16] Thielen FW, et al. Towards sustainability and affordability of expensive cell and gene therapies? Applying a cost-based pricing model to estimate prices for Libmeldy and Zolgensma. Cytotherapy. 2022;24(12):1245–58. 36216697 10.1016/j.jcyt.2022.09.002

[17] Nuijten M. Pricing Zolgensma—the world’s most expensive drug. J Mark Access Health Policy. 2022;10(1):2022353. 34992762 10.1080/20016689.2021.2022353 PMC8725676

[18] European Commission, Information and Notices. Official Journal of the European Union, 2020;63(C453).

[19] Committee for Medicinal Products for Human Use. Assessment report Oxlumo. Amsterdam: European Medicines Agency; 2020. Available from https://www.ema.europa.eu/en/documents/assessment-report/oxlumo-epar-public-assessment-report_en.pdf. Accessed 21 July 2022.

[20] van Woerden CS, et al. Primary hyperoxaluria type 1 in The Netherlands: prevalence and outcome. Nephrol Dial Transplant. 2003;18(2):273–9. 12543880 10.1093/ndt/18.2.273

[21] OxalEuropeRegistry. 2021.

[22] Cochat P. Primary hyperoxaluria type 1. Kidney Int. 1999;55(6):2533–47. 10354306 10.1046/j.1523-1755.1999.00477.x

[23] Cochat P, Groothoff J. Primary hyperoxaluria type 1: practical and ethical issues. Pediatr Nephrol. 2013;28(12):2273–81. 23494551 10.1007/s00467-013-2444-5

[24] Groothoff JW, et al. Clinical practice recommendations for primary hyperoxaluria: an expert consensus statement from ERKNet and OxalEurope. Nat Rev Nephrol. 2023;19(3):194–211. 36604599 10.1038/s41581-022-00661-1

[25] Milliner DS, et al. GeneReviews® [Internet]. In: Primary hyperoxaluria type 1. 2002 [updated 2022 Feb 10]: Seattle, W.A.

[26] Cochat P, et al. Primary hyperoxaluria type 1: indications for screening and guidance for diagnosis and treatment. Nephrol Dial Transplant. 2012;27(5):1729–36. 22547750 10.1093/ndt/gfs078

[27] van der Schans S, et al. A novel perspective on pharmaceutical R&D costs: opportunities for reductions. Expert Rev Pharmacoecon Outcomes Res. 2022;22(2):167–75. 34595997 10.1080/14737167.2022.1987219

[28] European Central Bank. Euro foreign exchange reference rates. Available from: https://www.ecb.europa.eu/stats/policy_and_exchange_rates/euro_reference_exchange_rates/html/index.en.html.

[29] The World Bank, Population, Total. Accessed May 9, 2023. Available from: https://data.worldbank.org/indicator/SP.POP.TOTL.

[30] Metry EL, et al. Long-term transplantation outcomes in patients with primary hyperoxaluria type 1 included in the European Hyperoxaluria Consortium (OxalEurope) Registry. Kidney International Reports; 2021. 10.1016/j.ekir.2021.11.006 PMC8821040 35155860

[31] Metry E. Determinants of kidney failure in Primary Hyperoxaluria type 1: findings of the European Hyperoxaluria Consortium. Accepted for publication in Kidney International reports; 2023. 10.1016/j.ekir.2023.07.025 PMC10577369 37849991

[32] Eurostat. Demography, population stock & balance. 2021 September 20, 2022. Available from: https://ec.europa.eu/eurostat/web/population-demography/demography-population-stock-balance/database.

[33] Alnylam Pharmaceuticals Inc. Annual SEC filings: form 10-K. 2020: Washington, D.C.

[34] Committee for Orphan Medicinal Products, Orphan Maintenance Assessment Report, in Oxlumo. 2020: Amsterdam. Available from: https://www.ema.europa.eu/en/documents/orphan-maintenance-report/oxlumo-orphan-maintenance-assessment-report-initial-authorisation_en.pdf. Accessed 21 July 2022.

[35] Alnylam Pharmaceuticals Inc. Annual SEC filings: form 10-K. 2010: Washington, D.C. Available from: https://www.sec.gov/edgar/browse/?CIK=1178670&owner=exclude. Accessed 21 July 2022.

[36] Alnylam Pharmaceuticals Inc. Annual SEC filings: form 10-K. 2011: Washington, D.C. Available from: https://www.sec.gov/edgar/browse/?CIK=1178670&owner=exclude. Accessed 21 July 2022.

[37] Alnylam Pharmaceuticals Inc. Annual SEC filing: form 10-K. 2012: Washington, D.C. Available from: https://www.sec.gov/edgar/browse/?CIK=1178670&owner=exclude. Accessed 21 July 2022.

[38] Alnylam Pharmaceuticals Inc. Annual SEC filing: form 10-K. 2013: Washington, D.C. Available from: https://www.sec.gov/edgar/browse/?CIK=1178670&owner=exclude. Accessed 21 July 2022.

[39] Alnylam Pharmaceuticals Inc. Annual SEC filings: form 10-K. 2014: Washington, D.C. Accessed 21 July 2022. Available from: https://www.sec.gov/edgar/browse/?CIK=1178670&owner=exclude.

[40] Alnylam Pharmaceuticals Inc. Annual SEC filings: form 10-K. 2015: Washington, D.C. Available from: https://www.sec.gov/edgar/browse/?CIK=1178670&owner=exclude. Accessed 21 July 2022.

[41] Alnylam Pharmaceuticals Inc. Annual SEC filings: form 10-K. 2016: Washington, D.C. Available from: https://www.sec.gov/edgar/browse/?CIK=1178670&owner=exclude. Accessed 21 July 2022.

[42] Alnylam Pharmaceuticals Inc. Annual SEC filings: form 10-K. 2017: Washington, D.C. Available from: https://www.sec.gov/edgar/browse/?CIK=1178670&owner=exclude. Accessed 21 July 2022.

[43] Alnylam Pharmaceuticals Inc. Annual SEC filings: form 10-K. 2018: Washington, D.C. Available from: https://www.sec.gov/edgar/browse/?CIK=1178670&owner=exclude. Accessed 21 July 2022.

[44] Alnylam Pharmaceuticals Inc. Annual SEC fillings: form 10-K. 2019: Washington, D.C. Available from: https://www.sec.gov/edgar/browse/?CIK=1178670&owner=exclude. Accessed 21 July 2022.

[45] Alnylam Pharmaceuticals Inc. Annual SEC filings: form 10-K. 2008: Washington, D.C. Available from: https://www.sec.gov/edgar/browse/?CIK=1178670&owner=exclude. Accessed 21 July 2022.

[46] Alnylam Pharmaceuticals Inc. Annual SEC filings: form 10-K. 2009: Washington, D.C. Available from: https://www.sec.gov/edgar/browse/?CIK=1178670&owner=exclude. Accessed 21 July 2022.

[47] Alnylam Pharmaceuticals Inc. Annual SEC filings: form 10-K. 2007: Washington, D.C. Available from: https://www.sec.gov/edgar/browse/?CIK=1178670&owner=exclude. Accessed 21 July 2022.

[48] Alnylam Pharmaceuticals Inc. Annual SEC filings: form 10-K. 2006: Washington, D.C. Accessed 21 July 2022. Available from: https://www.sec.gov/edgar/browse/?CIK=1178670&owner=exclude

[49] Alnylam Pharmaceuticals Inc. Annual SEC filings: form 10-K. 2002: Washington, D.C. Accessed 21 July 2022. Available from: https://www.sec.gov/edgar/browse/?CIK=1178670&owner=exclude

[50] Alnylam Pharmaceuticals Inc. Annual SEC filings: form 10-K. 2005: Washington, D.C. Accessed 21 July 2022. Available from: https://www.sec.gov/edgar/browse/?CIK=1178670&owner=exclude

[51] Alnylam Pharmaceuticals Inc. Annual SEC filings: form 10-K. 2004: Washington, D.C. Available from: https://www.sec.gov/edgar/browse/?CIK=1178670&owner=exclude. Accessed 21 July 2022.

[52] Alnylam Pharmaceuticals Inc. Annual SEC filings: form 10-K. 2003: Washington, D.C. Available from: https://www.sec.gov/edgar/browse/?CIK=1178670&owner=exclude. Accessed 21 July 2022.

[53] Michael M, et al. Lumasiran for advanced primary hyperoxaluria type 1: phase 3 ILLUMINATE-C trial. Am J Kidney Dis. 2023;81(2):145–55. 35843439 10.1053/j.ajkd.2022.05.012

[54] Garrelfs SF, et al. Lumasiran, an RNAi therapeutic for primary hyperoxaluria type 1. N Engl J Med. 2021;384(13):1216–26. 33789010 10.1056/NEJMoa2021712

[55] Hayes W, et al. Efficacy and safety of lumasiran for infants and young children with primary hyperoxaluria type 1: 12-month analysis of the phase 3 ILLUMINATE-B trial. Pediatr Nephrol. 2023;38(4):1075–86. 35913563 10.1007/s00467-022-05684-1 PMC9925547

[56] Hulton SA, et al. Randomized clinical trial on the long-term efficacy and safety of lumasiran in patients with primary hyperoxaluria type 1. Kidney Int Rep. 2022;7(3):494–506. 35257062 10.1016/j.ekir.2021.12.001 PMC8897294

[57] Eurostat. Population and population change statistics. 2021. Available from: https://ec.europa.eu/eurostat/statistics-explained/index.php?title=Population_and_population_change_statistics. Accessed 20 Sep 2022.

[58] Alnylam Pharmaceuticals I. Alnylam receives approval for OXLUMO™ (lumasiran) in the European Union for the treatment of primary hyperoxaluria type 1 in all age groups. 2020, Business Wire: Cambridge, MA. Available from: https://www.businesswire.com/news/home/20201119005667/en/. Accessed 5 Jan 2024.

[59] Jacobs J, Livestro D, Oosterwaal M. The cost of opportunity: a study on pharmaceutical R&D costs. Gupta strategists. 2019.

[60] Prasad V, Mailankody S. Research and development spending to bring a single cancer drug to market and revenues after approval. JAMA Intern Med. 2017;177(11):1569–75. 28892524 10.1001/jamainternmed.2017.3601 PMC5710275

[61] U.S. Securities and Exchange Commission, Edgar| Company filings. 2022. Available from: https://www.sec.gov/edgar/searchedgar/companysearch.html.

[62] Heine R, et al. Applying a cost-based pricing model for innovative cancer treatments subject to indication expansion: a case study for pembrolizumab and daratumumab. PLoS ONE. 2024;19(2):e0293264. 38300937 10.1371/journal.pone.0293264 PMC10833582

[63] Institut für Qualität und Wirtschaftlichkeit im Gesundheitswesen, Lumasiran (Hyperoxalurie)—Bewertung gemäß § 35a Abs. 1 Satz 11 SGB V. 2021: Köln, Germany. Accessed 23 Feb 2023. Available from: https://www.iqwig.de/download/g21-02_lumasiran_bewertung-35a-absatz-1-satz-11-sgb-v_v1-0.pdf.

[64] Baras AI, Baras AS, Schulman KA. Drug development risk and the cost of capital. Nat Rev Drug Discov. 2012;11(5):347–8. 22498751 10.1038/nrd3722

[65] Baum MA, et al. PHYOX2: a pivotal randomized study of nedosiran in primary hyperoxaluria type 1 or 2. Kidney Int. 2023;103(1):207–17. 36007597 10.1016/j.kint.2022.07.025

[66] Syed YY. Nedosiran: first approval. Drugs. 2023;83(18):1729–33. 38060091 10.1007/s40265-023-01976-4 PMC10803381

[67] Drug Pricing Lab. Curbing Biologic Drug Spending with P-quad. 2021. Available from: https://www.drugpricinglab.org/news/curbing-biologic-drug-spending-with-p-quad/. Accessed 6 Feb 2024.

[68] Médicins Sans Frontières, MSF Clinical Trial Transparency Policy. 2022. Available from: https://msfaccess.org/sites/default/files/2022-11/R%26D_MSF_Clinical%20Trial%20TransparencyPolicy_2022_ENG.pdf. Accessed 10 May 2023.

[69] Manders EA, van den Berg S, de Visser SJ, et al. Drug pricing models, no ‘one-size-fits-all’ approach: a systematic review and critical evaluation of pricing models in an evolving pharmaceutical landscape. Eur J Health Econ (2024). 10.1007/s10198-024-01731-w 39495345 10.1007/s10198-024-01731-w PMC12126323

