# Supplementary material for: Application of four pricing models for orphan medicines: a case study for lumasiran
Source: Orphanet J Rare Dis. 2024 Dec 23;19:485. doi: 10.1186/s13023-024-03446-w (PMC11667848; doi:10.1186/s13023-024-03446-w)
Supplement: Supplementary file 1 — Additional file1 (DOCX 90 KB) [file 13023_2024_3446_MOESM1_ESM.docx]

# Additional files

Application of four pricing models for orphan medicines: a case study for lumasiran

Overview of Additional files

[Additional files 1](#_Toc174991071)

[Additional file 1: Elaborate description and methods of the novel cancer pricing model 2](#_Toc174991072)

[Additional file 2: Elaborate description and methods of the AIM-model 3](#_Toc174991073)

[Additional file 3: Elaborate description and methods of the Discounted cash flow method 4](#_Toc174991074)

[Additional file 4: Elaborate description and methods of the real-option-rate-of-return method 6](#_Toc174991075)

[Additional file 5: Overview of research and development costs, cost of sold goods expenditures, and number of commercially treated patients derived from Alnylam Pharmaceuticals’ United States Security and Exchange Committee fillings from 2002 to 2020. US Dollars have been converted to Euros using the average 2021 exchange rate. 8](#_Toc174991076)

[Additional file 6: Patients per year for the discounted cash flow model. 9](#_Toc174991077)

[Additional file 6a: registry data for the number of prevalent patients 9](#_Toc174991078)

[Additional file 6b: Minimum scenario patients per year for the discounted cash flow model. 10](#_Toc174991079)

[Additional file 6c: Maximum scenario patients per year for the discounted cash flow model. 11](#_Toc174991080)

[Additional file 6d: Average scenario patients per year for the discounted cash flow model. 12](#_Toc174991081)

[Additional file 7: Cash flow calculations for the discounted cash flow model. 13](#_Toc174991082)

[Additional file 7a: Minimum scenario for the cash flow calculation for the discounted cash flow model. 13](#_Toc174991083)

[Additional file 7b: Maximum scenario for the cash flow calculation for the discounted cash flow model. 15](#_Toc174991084)

[Additional file 7c: Average scenario for the cash flow calculation for the discounted cash flow model. 17](#_Toc174991085)

[Additional file 8 : Constant patient numbers 18](#_Toc174991086)

[**References** 19](#_Toc174991087)

## Additional file 1: Elaborate description and methods of the novel cancer pricing model

Uyl-Den Groot & Löwenberg (2018) proposed a novel model for anticancer medicines based on transparency of actual costs and a profit margin related to the anticipated level of clinical benefit. [1]. Apart from anticancer medicines, the model can be applied to orphan medicines [1].

$average price per patient per year=\frac{R\&D\_cost}{Patients*Patent\_years\_left}+Medicine\_costs+Profit\_margin$ (EQ1)

The novel cancer pricing model (NCP-model) has the following input variables (**Table 1**):

- Research and development (R&D) costs: The NCP-model accounts for actual R&D costs [2]. We retrieved Alnylam Pharmaceuticals’ aggregated R&D costs from 2002 to 2020 from the United States (U.S.) Security and Exchange Commission (SEC) filings (€3.3 billion, see supplement 5 for a detailed overview) [3-21]. As of 2021, three Alnylam Pharmaceutical therapies have been approved [13]. For the maximum scenario, we divided the total aggregated R&D costs over this number of authorized products (€1.1 billion). For the minimum scenario, we accounted for an additional product (€8.3 million) (**Table 2**). Besides, we corrected this for the European Union (EU) population by using the EU contribution factor of 35,85%, congruent to the AIM model, as described below.
- Drug costs: The drug costs include marketing and sales and manufacturing [2]. NCPM estimates the marketing and sales costs at 30% of manufacturing costs [2]. Manufacturing costs are included in the aggregated Costs of Sold Goods (CoSG) in the SEC filings since the first commercially treated patient in 2018 [11-13]. The manufacturing costs per patient were calculated by dividing the total aggregated CoSG over the commercially treated patients reported in the SEC filings (€38,000, see Supplement 4) [11-13]. This should be interpreted with care due to two reasons. First, the CoSG may include other expenses apart from manufacturing costs [11-13]. Second, patients may also be treated in non-commercial settings, such as in clinical trials and compassionate use programs.
- Patients: The prevalence of primary hyperoxaluria type 1 (PH1) in the EU was estimated based on scientific literature [22] (2.9:1 million; minimum scenario) and the cases in the EU Member States in the OxalEuropeRegister [23] (1.6:1 million; maximum scenario, Supplement 5a). Treatment rates varied between 70% (minimum scenario) and 50% (maximum scenario) [24, 25].
- Patent years: Patents for lumasiran remain active until 2035 [13] (14 years, minimum scenario). As patents can cease or be appealed, we used the market exclusivity for orphan medicinal products (OMPs) that applies for ten years for the maximum scenario [26] (maximum scenario).
- Profit margin: The profit margin is linked to the clinical benefit and ranges between 20% (minimum scenario) and 40% (maximum scenario) [2].

## Additional file 2: Elaborate description and methods of the AIM-model

The AIM-model model was developed by the International Association of Mutual Benefit Societies (AIM) to create a “fair European maximum price calculation model” based on actual costs, a profit margin and an innovation bonus [2].

$average price per patient per year=\frac{R\&D\_cost}{Patients*Treatment\_duration}+Production\&Overhead\_cost+Sales\&Medical\_Information\_cost +Basic\_profit+Innovation_{bonus}$ (EQ2)

AIM-model has the following input variables (**Table 1**):

- R&D costs: AIM-model allows a lump sum for R&D spending, but companies are allowed to document that they invested more [28]. AIM-model suggested a minimum of €800 million for biotech medicines [28]. For the maximum scenario, we used Alnylam Pharmaceuticals’ SEC filings (€1.1 billion, see NCP-model in supplement 1) [3-13, 15-21]. Furthermore, AIM-model calculates the R&D costs attributable to the EU by utilizing the proportion of the EU population from the total population in developed countries (35.85%).
- Patients: The prevalence of PH1 in the EU was estimated based on scientific literature [22] (2.9 million:1 million; minimum scenario) and the cases in the EU Member States in the OxalEuropeRegister (1.6 million:1 million; maximum scenario) [23, 27]. Treatment rates varied between 70% (all patients but those that have undergone liver transplantation, minimum scenario) and 50% (nearly all patients apart from those that have undergone liver transplantation and are fully responsive to pyridoxine, maximum scenario) [24, 25], and the market share varied between 100% (no competitors, minimum scenario) and 50% (competitor(s), maximum scenario).
- Treatment duration: AIM-model proposed to link treatment duration to patent years. Patents remain active until 2035 [13] (14 years, minimum scenario). However, for the maximum scenario, market exclusivity for orphan medicinal products is limited to 10 years [26].
- Production and overhead costs: AIM-model suggests €250 per month (minimum scenario) for chemical OMPs and €750 per month (maximum scenario) for biological OMPs [27] per patient per month.
- Sales and medical information: AIM-model proposes 20% of R&D costs for sales and medical information, gradually reduced. We used 20% in the maximum and 15% in the minimum scenario.
- Basic profit: AIM-model proposes an 8% basic profit margin [27].
- Innovation bonus: The innovation bonus is linked to the treatment value and ranges between 5% (minimum scenario) and 40% (maximum scenario) [27].

## Additional file 3: Elaborate description and methods of the Discounted cash flow method

Nuijten & Vis (2016) developed a method to evaluate and valuate innovative medicines [3]. This method calculates a price from the investor’s perspective using the business valuation theory [3]. The valuation is based on the discounted cash flow (DCF) method which uses Free Cash Flows (FCF) and the required Cost of Capital (CoC) [3]. This means that the cash flows consisting of costs (R&D costs, production costs and marketing costs) and revenues are discounted per year to calculate a present value. Next, the price at which the Net Present Value (NPV) equals zero is calculated [3].

$NPV=\sum_{0}^{n\_max} \frac{{CF}_{n}}{{CoC}^{n}}$ (EQ3)

${CF}_{n}=Revenue_{n}-\left( R\&D \_costs\_for\_EU_{n}+Production\_costs_{n}+Marketing\_costs_{n} \right)=Revenue_{n}-(0,3585*R\&D\_costs_{n} +0,4*Revenue_{n})$(EQ4)

${Patients}_{n}=\left( \left( Prevalence*EU\_population \right)+\sum_{0}^{n} \left( Incidence*{EU\_population}_{n} \right) \right)*P_{Response}*P_{Fail}*P_{AdverseEvents-stop}*P_{Contraindication}*P_{PrescriptionRestriction}*P_{Uptake\_n}$ (EQ5)

${Revenue}_{n}={Patients}_{n}*average price per patient per year$ (EQ6)

The model uses the following input variables (**Table 3**):

- R&D costs: According to the DCF-model, a lump sum of $700 million should stimulate efficient use of resources (Additional File 7) [29]. Similarly, to the AIM-model and NCP-model, we used an EU contribution factor of 35,85%, see above.
- Cost of Capital: 9% for pharmaceuticals (minimum scenario) and 12% for biotech (maximum scenario) applies [29].
- Costs: The DCF-model suggests the cost of medicines, including production and sales, to be at 40% of revenues [29].
- Patients: Prevalence and incidence were estimated based on data from the scientific literature (prevalence of 2.9 million:1 million, incidence 0.15 million:1 million, minimum scenario) and the OxalEuropeRegistry (prevalence 1.6 million:1 million, incidence 0.18 million:1 million, maximum scenario) [22]. Apart from prevalence and incidence, DCF-model proposes using actual data from clinical trials on response, failures, adverse events, contraindications, prescription restrictions (Table 2), and uptake curves to estimate the number of patients treated (Additional File 7) [29]. These data were derived from the European Public Assessment Reports (EPAR) on lumasiran, www.clinicaltrials.gov, and scientific literature [24-26, 30-35]. Minimum and maximum estimates were supported by expert opinion.
- Treatment duration: Treatment duration is linked to patent years. In this case, we estimated the duration at 10-14 years [13].

In addition to this, Nuijten & Capri (2020) proposed an innovation premium to calculate an upper price limit [36]. This innovation premium consists of monetary (cost savings) and non-monetary values (QALYs) for society [36]. Cost savings include direct medical costs, direct non-medical costs, and indirect costs [36]. As expected, QALYs and cost savings are currently unknown; we chose to use a fixed percentage as an innovation premium ranging between 5 to 15 percent for the minimum and maximum scenarios.

## Additional file 4: Elaborate description and methods of the real-option-rate-of-return method

Building further on the Gupta strategists' report [4], which calculates the total R&D costs per New Molecular Entity (NME), van der Schans et al. (2022) have developed a comprehensive economic model used to determine medicine prices [5]. It stands out by explicitly considering both production and research and development costs, encompassing out-of-pocket costs, failure costs, and CoC. Besides, it includes the weighted average cost of capital (WACC) and a deflator for the gross domestic product (GDP). By examining the full range of expenses, the model aims to gain a precise understanding of R&D costs. Pricing is determined based on these detailed costs, incorporating a set rate of return (RoR).

$Average price per patient per year = \left( \frac{R\&D costs}{Average number of patients per year*patent period}+production costs per patient per year \right)*\left( 1+profit margin \right)$  (EQ7)

$R\&D costs=Out of pocket costs+Failure costs+Cost of Capital$ (EQ8)

$$Failure costs=Out of pocket costs per phase*\frac{1}{cummulative success rate per phase}$$

 (EQ9)

$$Cost of Capital= \left( R\&D costs* \left( 1+WACC \right) \right)^{average time until approval} * \left( 1-GDP deflator \right)^{average time until approval}$$

(EQ10)

The model uses the following input variables

- Patients: See NCP-model
- Patent years: See NCP-model
- Production costs: See NCP-model
- R&D costs: We made the assumption that the R&D costs parameter in the calculation signifies total project investments (adjusted for the CoC), while the R&D parameter in the CoC calculation represents the net R&D investments (including Out-of-pocket costs and Failure costs). The latter were already known from the SEC filings and could be used. Similarly to the previously described models, we incorporated an EU contribution factor of 35,85%.
- WACC: Gupta strategists provide a comprehensive overview of R&D costs associated with New Molecular Entities (NMEs). This is based on historical data derived from 70,000 trials registered in ClinicalTrials.gov, along with a meta-analysis of existing literature that explores these associations. The findings present an overview of the Weighted Average Cost of Capital (WACC) within the pharmaceutical industry up to the year 2017. The underlying assumption is that the WACC remains consistent across all types of medicines and diseases. In the maximum scenario, a WACC of 14% is considered, while in the minimum scenario, a WACC of 8% is adopted. These percentages represent the highest and lowest observed values in the historical data leading up to 2017. In the average scenario, an average of 11% is assumed.
- GDP deflator: The gross domestic product (GDP) deflator is a measure of the average price change of all goods and services produced in an economy, indicating the inflation or deflation of the overall price level. This metric is computed by dividing the nominal GDP by the real GDP and then multiplying the result by 100 to express it as an index. The GDP deflator for EU countries in 2022 was 6.5%, which will be utilized for the maximum scenario [6]. In the minimum scenario, an index of 1.9% will be employed, corresponding to the GDP deflator in 2020—the year of approval. For the average scenario, an index of 4.2% will be applied.
- Average time until approval: In a span of six years, Alnylam successfully navigated lumasiran from compound identification to regulatory approval [7]. This will be used for the minimum scenario. The Gupta strategist report, which forms the foundation for this model, assumes a duration of 10-15 years [4]. Therefore, a time until approval of 15 years has been chosen for the maximum scenario.
- Profit margin: The fixed percentages of the DCFM will be used which ranges between 5% (minimum scenario) and 15% (maximum scenario). The choice was made to use similar precentages as the DCF-model also accounts for the CoC.

## Additional file 5: Overview of research and development costs, cost of sold goods expenditures, and number of commercially treated patients derived from Alnylam Pharmaceuticals’ United States Security and Exchange Committee fillings from 2002 to 2020. US Dollars have been converted to Euros using the average 2021 exchange rate.

| **Year** | **Aggregated R&D costs of Alnylam** | **Aggregated Cost of Sold Goods of Alnylam** | **Aggregated number of commercially treated patients on Oxlumo, Onpattro and Givlaari** |
| --- | --- | --- | --- |
| 2002 | €2.550.000 | € - | - |
| 2003 | € 11.050.000 | € - | - |
| 2004 | € 21.250.000 | € - | - |
| 2005 | € 29.750.000 | € - | - |
| 2006 | € 41.650.000 | € - | - |
| 2007 | € 102.850.000 | € - | - |
| 2008 | € 82.450.000 | € - | - |
| 2009 | € 92.650.000 | € - | - |
| 2010 | € 90.426.400 | € - | - |
| 2011 | € 84.400.750 | € - | - |
| 2012 | € 73.583.650 | € - | - |
| 2013 | € 96.013.450 | € - | - |
| 2014 | € 161.711.650 | € - | - |
| 2015 | € 235.020.750 | € - | - |
| 2016 | € 325.033.200 | € - | - |
| 2017 | € 332.039.750 | € - | - |
| 2018 | € 429.607.000 | € 1.531.700 | 56 |
| 2019 | € 556.849.900 | € 21.302.700 | 750 |
| 2020 | € 556.596.150 | € 66.344.200 | 1.552 |
| **Total** | **€ 3.325.479.650** | **€ 89.178.600** | **2.357** |

## Additional file 6: Patients per year for the discounted cash flow model.

### Additional file 6a: registry data for the number of prevalent patients

| **Country** | **Cases** | **Population** | **EU Member State** |
| --- | --- | --- | --- |
| Albania | 2 | 2800000 | Yes |
| Algeria | 3 | 44200000 | No |
| Austria | 10 | 9000000 | Yes |
| Azerbaijan | 1 | 10100000 | No |
| Belgium | 24 | 11600000 | Yes |
| Denmark | 13 | 5900000 | Yes |
| Finland | 4 | 5500000 | Yes |
| France | 173 | 67800000 | Yes |
| Germany | 148 | 83200000 | Yes |
| Greece | 7 | 10600000 | Yes |
| Italy | 88 | 59100000 | Yes |
| Lithuania | 1 | 2800000 | Yes |
| Macedonia | 2 | 2100000 | No |
| Netherlands | 95 | 17500000 | Yes |
| Norway | 17 | 5400000 | No |
| Poland | 13 | 37800000 | Yes |
| Portugal | 2 | 10300000 | Yes |
| Russia | 8 | 143400000 | No |
| Serbia | 2 | 6800000 | No |
| Slovinia | 1 | 2100000 | Yes |
| Spain | 28 | 47400000 | Yes |
| Sweden | 7 | 10400000 | Yes |
| Switzerland | 12 | 8700000 | No |
| Syria | 2 | 21300000 | No |
| Turkey | 19 | 84800000 | No |
| United Kingdom | 183 | 67300000 | No |

### Additional file 6b: Minimum scenario patients per year for the discounted cash flow model.

| **Year** | **Patients per year** | **uptake** | **Number of sales** |
| --- | --- | --- | --- |
| 1 | 713 | 0,0 | 0 |
| 2 | 770 | 0,2 | 139 |
| 3 | 827 | 0,3 | 264 |
| 4 | 883 | 0,4 | 371 |
| 5 | 941 | 0,5 | 452 |
| 6 | 998 | 0,5 | 499 |
| 7 | 1055 | 0,5 | 521 |
| 8 | 1112 | 0,5 | 529 |
| 9 | 1169 | 0,4 | 520 |
| 10 | 1226 | 0,4 | 492 |
| 11 | 1283 | 0,3 | 443 |
| 12 | 1340 | 0,3 | 372 |
| 13 | 1397 | 0,2 | 276 |
| 14 | 1454 | 0,1 | 153 |
| 15 | 1511 | 0,0 | 0 |

### Additional file 6c: Maximum scenario patients per year for the discounted cash flow model.

| **Year** | **Patients per year** | **Uptake** | **Number of sales** |
| --- | --- | --- | --- |
| 1 | 250 | 0,0 | 0 |
| 2 | 302 | 0,2 | 54 |
| 3 | 353 | 0,3 | 113 |
| 4 | 404 | 0,4 | 170 |
| 5 | 455 | 0,5 | 219 |
| 6 | 507 | 0,5 | 253 |
| 7 | 558 | 0,5 | 268 |
| 8 | 609 | 0,4 | 256 |
| 9 | 661 | 0,3 | 211 |
| 10 | 712 | 0,2 | 128 |
| 11 | 763 | 0,0 | 0 |
| 12 | 814 | 0,0 | 0 |
| 13 | 866 | 0,0 | 0 |
| 14 | 917 | 0,0 | 0 |
| 15 | 968 | 0,0 | 0 |

### Additional file 6d: Average scenario patients per year for the discounted cash flow model.

| **Year** | **Patients per year** | **Uptake** | **Number of sales** |
| --- | --- | --- | --- |
| 1 | 453 | 0 | 0 |
| 2 | 507 | 0,2 | 91 |
| 3 | 562 | 0,3 | 180 |
| 4 | 617 | 0,4 | 259 |
| 5 | 671 | 0,5 | 322 |
| 6 | 726 | 0,5 | 363 |
| 7 | 781 | 0,5 | 380 |
| 8 | 834 | 0,4 | 374 |
| 9 | 890 | 0,4 | 340 |
| 10 | 945 | 0,3 | 275 |
| 11 | 1000 | 0,2 | 173 |
| 12 | 1055 | 0,0 | 30 |
| 13 | 1109 | 0,0 | 0 |
| 14 | 1164 | 0,0 | 0 |
| 15 | 1219 | 0,0 | 0 |

## Additional file 7: Cash flow calculations for the discounted cash flow model.

### Additional file 7a: Minimum scenario for the cash flow calculation for the discounted cash flow model.

R&D costs are based on the lump sum for R&D costs based on Nuijten & Vis (2016) [3]. US Dollars were converted to Euros and were multiplied by 35,85% to calculate the R&D costs attributable to the EU. Sales costs are 40% of revenues [3]. Revenue is based on the number of sales multiplied by the price at which the net present value is zero. The net present value is the sum of all present values. The present values are calculated by discounting the cash flows by the cost of capital. A cost of capital of 9% for pharmaceuticals is used [3]. Year 0 is the year in which there is no discounting yet. Year 8 is the year of the marketing authorization application.

| **Year** | **R&D costs for EU** | **Sales costs** | **Revenues** | **Cash flows** | **Present value** | **Cumulative present value** |
| --- | --- | --- | --- | --- | --- | --- |
| 0 | € 66.402.625 | € - | € - | € -66.402.625 | € -66.402.625 | € -66.402.625 |
| 1 | € 12.809.115 | € - | € - | € -12.809.115 | € -11.751.482 | € -78.154.107 |
| 2 | € 12.809.115 | € - | € - | € -12.809.115 | € -10.781.176 | € -88.935.283 |
| 3 | € 21.734.511 | € - | € - | € -21.734.511 | € -16.783.030 | € -105.718.313 |
| 4 | € 21.734.511 | € - | € - | € -21.734.511 | € -15.397.275 | € -121.115.588 |
| 5 | € 7.516.012 | € - | € - | € -7.516.012 | € -4.884.892 | € -126.000.480 |
| 6 | € 7.516.012 | € - | € - | € -7.516.012 | € -4.481.552 | € -130.482.032 |
| 7 | € 7.516.012 | € - | € - | € -7.516.012 | € -4.111.516 | € -134.593.548 |
| 8 | € - | € - | € - | € - | € - | € -134.593.548 |
| 9 | € 1.120.633 | € - | € - | € -1.120.633 | € -515.970 | € -135.109.518 |
| 10 | € 1.120.633 | € 9.614.817 | € 24.037.041 | € 13.301.592 | € 5.618.736 | € -129.490.782 |
| 11 | € 1.120.633 | € 18.358.237 | € 45.895.593 | € 26.416.723 | € 10.237.348 | € -119.253.434 |
| 12 | € 1.120.633 | € 25.755.800 | € 64.389.501 | € 37.513.068 | € 13.337.198 | € -105.916.236 |
| 13 | € - | € 31.333.045 | € 78.332.613 | € 46.999.568 | € 15.330.255 | € -90.585.980 |
| 14 | € - | € 34.615.510 | € 86.538.776 | € 51.923.266 | € 15.537.854 | € -75.048.127 |
| 15 | € - | € 36.140.674 | € 90.351.684 | € 54.211.010 | € 14.882.985 | € -60.165.142 |
| 16 | € - | € 36.664.694 | € 91.661.736 | € 54.997.041 | € 13.852.092 | € -46.313.050 |
| 17 | € - | € 36.041.134 | € 90.102.834 | € 54.061.700 | € 12.492.209 | € -33.820.842 |
| 18 | € - | € 34.123.553 | € 85.308.883 | € 51.185.330 | € 10.850.969 | € -22.969.872 |
| 19 | € - | € 30.765.514 | € 76.913.785 | € 46.148.271 | € 8.975.362 | € -13.994.510 |
| 20 | € - | € 25.820.578 | € 64.551.445 | € 38.730.867 | € 6.910.783 | € -7.083.727 |
| 21 | € - | € 19.142.306 | € 47.855.765 | € 28.713.459 | € 4.700.338 | € -2.383.389 |
| 22 | € - | € 10.584.260 | € 26.460.649 | € 15.876.389 | € 2.384.343 | € ~ 0 |
| 23 | € - | € - | € - | € - | € - |  |

### Additional file 7b: Maximum scenario for the cash flow calculation for the discounted cash flow model.

R&D costs are based on the lump sum for R&D costs based on Nuijten & Vis (2016) (Nuijten and Vis 2016). US Dollars were converted to Euros using the average 2021 conversion rate and were multiplied by 35,85% to calculate the R&D costs attributable to the EU. Sales costs are 40% of revenues (Nuijten and Vis 2016). Revenue is based on the number of sales multiplied by the price at which the net present value is zero. The net present value is the sum of all present values. The present values are calculated by discounting the cash flows by the cost of capital. A cost of capital of 12% for biotech is used (Nuijten and Vis 2016). Year 0 is the year in which there is no discounting yet. Year 8 is the year of the marketing authorization application.

| Year | R&D costs for EU | Sales costs | Revenues | Cash flows | Present value | Cumulative present value |
| --- | --- | --- | --- | --- | --- | --- |
| 0 | € 66.402.625 | € - | € - | € -66.402.625 | €-66.402.625 | € -66.402.625 |
| 1 | € 12.809.115 | € - | € - | € -12.809.115 | €-11.436.710 | € -77.839.335 |
| 2 | € 12.809.115 | € - | € - | € -12.809.115 | €-10.211.348 | € -88.050.683 |
| 3 | € 21.734.511 | € - | € - | € -21.734.511 | €-15.470.195 | € -103.520.879 |
| 4 | € 21.734.511 | € - | € - | € -21.734.511 | €-13.812.674 | € -117.333.553 |
| 5 | € 7.516.012 | € - | € - | € -7.516.012 | € -4.264.787 | € -121.598.340 |
| 6 | € 7.516.012 | € - | € - | € -7.516.012 | € -3.807.845 | € -125.406.185 |
| 7 | € 7.516.012 | € - | € - | € -7.516.012 | € -3.399.862 | € -128.806.047 |
| 8 | € - | € - | € - | € - | € - | € -128.806.047 |
| 9 | € 1.120.633 | € - | € - | € -1.120.633 | € -404.111 | € -129.210.159 |
| 10 | € 1.120.633 | € 14.124.696 | € 35.311.741 | € 20.066.412 | € 6.460.848 | € -122.749.311 |
| 11 | € 1.120.633 | € 29.379.587 | € 73.448.968 | € 42.948.748 | € 12.346.739 | € -110.402.572 |
| 12 | € 1.120.633 | € 44.163.792 | € 110.409.479 | € 65.125.055 | € 16.715.979 | € -93.686.593 |
| 13 | € - | € 56.876.429 | € 142.191.072 | € 85.314.643 | € 19.551.914 | € -74.134.679 |
| 14 | € - | € 65.916.617 | € 164.791.544 | € 98.874.926 | € 20.231.769 | € -53.902.910 |
| 15 | € - | € 69.683.477 | € 174.208.692 | € 104.525.215 | € 19.096.366 | € -34.806.544 |
| 16 | € - | € 66.576.126 | € 166.440.314 | € 99.864.188 | € 16.290.012 | € -18.516.531 |
| 17 | € - | € 54.993.683 | € 137.484.208 | € 82.490.525 | € 12.014.278 | € -6.502.253 |
| 18 | € - | € 33.335.268 | € 83.338.171 | € 50.002.902 | € 6.502.357 | € ~0 |
| 19 | € - | € - | € - | € - | € - | € - |
| 20 | € - | € - | € - | € - | € - | € - |
| 21 | € - | € - | € - | € - | € - | € - |
| 22 | € - | € - | € - | € - | € - | € - |
| 23 | € - | € - | € - | € - | € - | € - |

### Additional file 7c: Average scenario for the cash flow calculation for the discounted cash flow model.

R&D costs are based on the lump sum for R&D costs based on Nuijten & Vis (2016) (Nuijten and Vis 2016). US Dollars were converted to Euros using the average 2021 conversion rate and were multiplied by 35,85% to calculate the R&D costs attributable to the EU. Sales costs are 40% of revenues (Nuijten and Vis 2016). Revenue is based on the number of sales multiplied by the price at which the net present value is zero. The net present value is the sum of all present values. The present values are calculated by discounting the cash flows by the cost of capital. An average cost of capital of 10,5% is used (Nuijten and Vis 2016). Year 0 is the year in which there is no discounting yet. Year 8 is the year of the marketing authorization application.

| Year | R&D costs for EU | Sales costs | Revenues | Cash flows | Present value | Cumulative present value |
| --- | --- | --- | --- | --- | --- | --- |
| 0 | € 66.402.625 | € - | € - | € -66.402.625 | € -66.402.625 | € -66.402.625 |
| 1 | € 12.809.115 | € - | € - | € -12.809.115 | € -11.591.960 | € -77.994.584 |
| 2 | € 12.809.115 | € - | € - | € -12.809.115 | € -10.490.461 | € -88.485.046 |
| 3 | € 21.734.511 | € - | € - | € -21.734.511 | € -16.108.794 | € -104.593.840 |
| 4 | € 21.734.511 | € - | € - | € -21.734.511 | € -14.578.094 | € -119.171.934 |
| 5 | € 7.516.012 | € - | € - | € -7.516.012 | € -4.562.218 | € -123.734.152 |
| 6 | € 7.516.012 | € - | € - | € -7.516.012 | € -4.128.704 | € -127.862.856 |
| 7 | € 7.516.012 | € - | € - | € -7.516.012 | € -3.736.384 | € -131.599.240 |
| 8 | € - | € - | € - | € - | € - | € -131.599.240 |
| 9 | € 1.120.633 | € - | € - | € -1.120.633 | € -456.250 | € -132.055.490 |
| 10 | € 1.120.633 | € 12.507.760 | € 31.269.401 | € 17.641.008 | € 6.499.809 | € -125.555.681 |
| 11 | € 1.120.633 | € 24.634.656 | € 61.586.640 | € 35.831.351 | € 11.947.530 | € -113.608.151 |
| 12 | € 1.120.633 | € 35.481.198 | € 88.702.994 | € 52.101.164 | € 15.721.721 | € -97.886.430 |
| 13 | € - | € 44.147.897 | € 110.369.741 | € 66.221.845 | € 18.083.882 | € -79.802.548 |
| 14 | € - | € 49.735.263 | € 124.338.158 | € 74.602.895 | € 18.436.725 | € -61.365.823 |
| 15 | € - | € 52.083.329 | € 130.208.323 | € 78.124.994 | € 17.472.531 | € -43.893.291 |
| 16 | € - | € 51.239.414 | € 128.098.535 | € 76.859.121 | € 15.556.037 | € -28.337.254 |
| 17 | € - | € 46.614.964 | € 116.537.409 | € 69.922.446 | € 12.807.309 | € -15.529.946 |
| 18 | € - | € 37.621.423 | € 94.053.559 | € 56.432.135 | € 9.354.175 | € -6.175.771 |
| 19 | € - | € 23.670.239 | € 59.175.597 | € 35.505.358 | € 5.326.116 | € -849.655 |
| 20 | € - | € 4.172.855 | € 10.432.138 | € 6.259.283 | € 849.726 | € ~0 |
| 21 | € - | € - | € - | € - | € - | € - |
| 22 | € - | € - | € - | € - | € - | € - |
| 23 | € - | € - | € - | € - | € - | € - |

# Additional file 8 : Constant patient numbers

This additional file presents the pricing outcomes of the NCP-, AIM-, DCF-, and ROROR-model when the patient number is kept constant across all models. For both minimum and maximum scenarios, 1,000 patients were inserted. Input factors related to estimating the actual number of patients, such as treatment rates and market shares, were omitted.
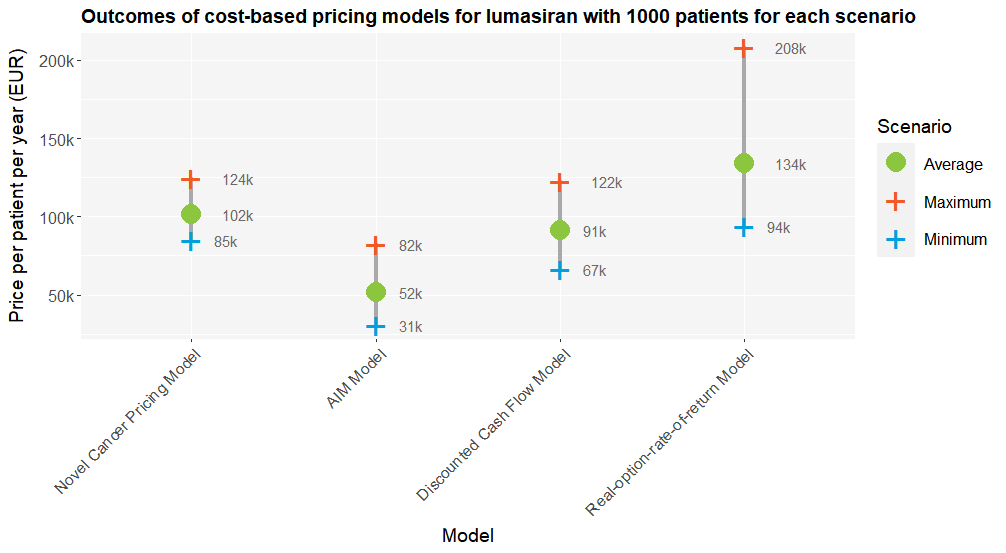


**References**

1. Uyl-de Groot, C.A. and B. Löwenberg, *Sustainability and affordability of cancer drugs: a novel pricing model.* Clinical Oncology, 2018. **15**: p. 405-406.

2. The International Association of Mutual Benefit Societies (AIM), *AIM Proposes to Establish a European Drug Pricing Model for Fair and Transparant Prices for Accessible Pharmaceutical Innovations*. 2019, The International Association of Mutual Benefit Societies

3. Nuijten, M. and J. Vis, *Evaluation and valuation of innovative medicinal products.* Journal of Rare Diseases Research & Treatment, 2016. **2**(1): p. 1-11.

4. Jacobs, J., D. Livestro, and M. Oosterwaal, *The cost of opportunity: a study on pharmaceutical R&D costs. Gupta strategists.* 2019.

5. van der Schans, S., et al., *A novel perspective on pharmaceutical R&D costs: opportunities for reductions.* Expert Rev Pharmacoecon Outcomes Res, 2022. **22**(2): p. 167-175.

6. The World Bank. *Inflation, GDP deflator (annual %) - European Union*. 2024 [cited 2024 January 5]; Available from: <https://data.worldbank.org/indicator/NY.GDP.DEFL.KD.ZG?locations=EU>.

7. Alnylam Pharmaceuticals, I., *Alnylam Receives Approval for OXLUMO™ (lumasiran) in the European Union for the Treatment of Primary Hyperoxaluria Type 1 in All Age Groups*. 2020, Business Wire: Cambridge, MA.
